# How do necrotic cells expose phosphatidylserine to attract their predators—What’s unique and what’s in common with apoptotic cells

**DOI:** 10.3389/fcell.2023.1170551

**Published:** 2023-04-05

**Authors:** Yoshitaka Furuta, Zheng Zhou

**Affiliations:** Verna and Marrs McLean Department of Biochemistry and Molecular Biology, Baylor College of Medicine, Houston, TX, United States

**Keywords:** apoptotic cells, necrotic cells, phagocytosis, phosphatidylserine, flippases, phospholipid scramblases, Ca^2+^, caspase

## Abstract

Phosphatidylserine (PS) is a lipid component of the plasma membrane. It is asymmetrically distributed to the inner leaflet in live cells. In cells undergoing apoptosis, phosphatidylserine is exposed to the outer surfaces. The exposed phosphatidylserine acts as an evolutionarily conserved “eat-me” signal that attracts neighboring engulfing cells in metazoan organisms, including the nematode *Caenorhabditis elegans*, the fruit fly *Drosophila melanogaster*, and mammals. During apoptosis, the exposure of phosphatidylserine to the outer surface of a cell is driven by the membrane scramblases and flippases, the activities of which are regulated by caspases. Cells undergoing necrosis, a kind of cell death frequently associated with cellular injuries and morphologically distinct from apoptosis, were initially believed to allow passive exposure of phosphatidylserine through membrane rupture. Later studies revealed that necrotic cells actively expose phosphatidylserine before any rupture occurs. A recent study in *C. elegans* further reported that the calcium ion (Ca^2+^) plays an essential role in promoting the exposure of phosphatidylserine on the surfaces of necrotic cells. These findings indicate that necrotic and apoptotic cells, which die through different molecular mechanisms, use common and unique mechanisms for promoting the exposure of the same “eat me” signal. This article will review the mechanisms regulating the exposure of phosphatidylserine on the surfaces of necrotic and apoptotic cells and highlight their similarities and differences.

## Introduction

The plasma membrane of eukaryotic cells is composed of the lipid bilayer and various membrane proteins. The lipid bilayer comprises glycerophospholipids, sphingomyelins, and cholesterol (Harayama and Riezman, 2018). Phosphatidylserine (PS), one type of glycerophospholipids, is a major component of the plasma membrane. In living cells, PS is almost exclusively localized to the inner leaflet of the plasma membrane (Balasubramanian and Schroit, 2003). PS exposure is observed when cells undergo apoptosis, a type of cell death that features cell shrinkage, chromatin condensation, nuclear DNA fragmentation, and a well-maintained plasma membrane integrity (Balasubramanian and Schroit, 2003; Kroemer et al., 2009). PS presented on the surfaces of apoptotic cells is an “eat-me” signal that attracts phagocytes to engulf apoptotic cells (Balasubramanian and Schroit, 2003; Segawa and Nagata, 2015). PS exposure also occurs in living cells under various physiological conditions. For example, mammalian platelets activated by vascular injuries expose PS, which recruits blood coagulation factors, and initiates blood clotting (Bevers et al., 1983; Dachary-Prigent et al., 1995). The exposure of PS on living cells and its biological significance have been extensively reviewed in another article (Shin and Takatsu, 2020) and is not covered here. Necrosis is another type of cell death that displays cell and organelle swelling, excessive intracellular membranes, and the subsequent rupture of intracellular and plasma membranes (Kroemer et al., 2009). Various conditions induce necrosis in cells, such as extreme temperature change, physical injury, hypoxia, hypo-osmotic shock, bacterial infection, ligands of transmembrane receptors such as the death receptors, and Ca^2+^ excitotoxicity (McCall, 2010; Moquin and Chan, 2010; Vlachos and Tavernarakis, 2010; Zhou and Yuan, 2014). Necrosis is closely associated with various diseases such as stroke, chronic inflammation, cancer, and neural and retinal degeneration (Yamashima, 2004; Noch and Khalili, 2009; Challa and Chan, 2010; Whelan et al., 2010; Nikoletopoulou and Tavernarakis, 2014; Shan et al., 2018; Zhang et al., 2023). For example, the hyperexcitation of neurons or glial cells induced by the constitutively active ion channels and related proteins causes excitotoxic necrosis (Noch and Khalili, 2009; Lai et al., 2014). Excitotoxic necrosis is a leading cause of neuronal damage in the brain ischemia (Tymianski, 2011; Lai et al., 2014). It also contributes to various aging-associated neurodegenerative disorders (Martin, 1999). Like apoptotic cells, necrotic cells are also engulfed and degraded by phagocytes (Hall et al., 1997; Krysko et al., 2006a; Poon et al., 2010b; Li et al., 2015; Schwegler et al., 2015). Efficient clearance of apoptotic and necrotic cells is essential for tissue homeostasis, tissue repair, and the suppression of harmful inflammatory and auto-immune responses caused by the contents of the dying cells during animal development and adulthood (Krysko et al., 2006a; Poon et al., 2010b; Poon et al., 2014). In addition, clearance of necrotic and apoptotic neurons helps to resolve the wounded area and facilitates tissue regeneration and the recovery from brain injury (Martin, 1999; Tymianski, 2011; Lai et al., 2014; Tovar-y-Romo et al., 2016; Tremblay et al., 2019). On the other hand, microglial cells, the professional phagocytes in the central nervous system, may also be activated by degenerating neurons and further enhance neural inflammation (Apolloni et al., 2014; Tremblay et al., 2019; Butler et al., 2021). Therefore, identifying cell clearance mechanisms is highly relevant to human health.

How necrotic cells are recognized and engulfed remained elusive for a long time. PS has been reported to serve as an “eat me” signal to recruit phagocytes for necrotic cells (Brouckaert et al., 2004; Schwegler et al., 2015; Budai et al., 2019). Whether the intracellular mechanisms that drive PS exposure are common or different between apoptotic and necrotic cells remains largely an unanswered question. Although besides PS, other “eat me” signals that attract phagocytes to necrotic cells were also identified (Gaipl et al., 2001; Böttcher et al., 2006; Poon et al., 2010a), this article will focus on PS-exposure mechanisms. Here we review the recent discoveries regarding the molecular mechanisms driving PS exposure on the surfaces of necrotic cells and how these mechanisms contribute to the phagocytosis of necrotic cells. Furthermore, we will compare these newly identified mechanisms to that driving PS exposure on the surfaces of apoptotic cells and discuss how these findings shed light on the roles of caspase 3 and Ca^2+^ in regulating PS exposure and dying cell-clearance.

## PS on the surfaces of both apoptotic and necrotic cells acts as an “eat me” signal that attracts phagocytes

The molecular mechanisms of PS-dependent phagocytosis of apoptotic cells were extensively reviewed previously (Elliott and Ravichandran, 2016; Nagata, 2018; Atkin-Smith, 2021). In summary, in mammals, PS exposed on apoptotic cells is recognized by phagocytic receptors *via* two alternative mechanisms: phagocytic receptors such as Tim-1 and Tim-4, stabilin 1 and 2, and BAI1 on neighboring engulfing cells directly interact with the PS molecules on the surfaces of apoptotic cells to trigger phagocytosis (Miyanishi et al., 2007; Park et al., 2007; Ichimura et al., 2008; Park et al., 2008; Park et al., 2009). On the other hand, some phagocytotic receptors recognize PS indirectly *via* binding to the bridging molecules secreted into the extracellular space to interact with PS. For example, the α_V_β_3_ integrin binds PS *via* the bridging protein MFG-E8 (Hanayama et al., 2002), and the Tryo3–Axl–Mer (TAM) family of receptors interact with PS *via* the bridging molecules Protein S or GAS6 in mammalian cells (Rothlin et al., 2015). PS on the surfaces of dying cells is directly recognized by the phagocytic receptor CED-1 in *Caenorhabditis elegans* (Zhou et al., 2001b; Li et al., 2015) and by Draper, the homolog of CED-1 in *Drosophila* (MacDonald et al., 2006; Tung et al., 2013). CED-1 was also proposed to interact with PS through TTR-52, a bridging molecule (Wang et al., 2010), indicating that it can use both mechanisms to recognize apoptotic cells. In the brain of mammals, microglia and astrocytes are known to engulf and degrade various extracellular materials (Uddin and Lim, 2022). MEGF10, the mammalian CED-1 ortholog, is expressed in astrocytes where it functions to phagocytose apoptotic cells (Scheib et al., 2012; Iram et al., 2016), synapses (Chung et al., 2013; Lee et al., 2021), and amyloid *β* aggregates (Singh et al., 2010; Fujita et al., 2020), demonstrating the functional conservation among the CED-1 family of proteins in phagocytosis.

**FIGURE 1:**
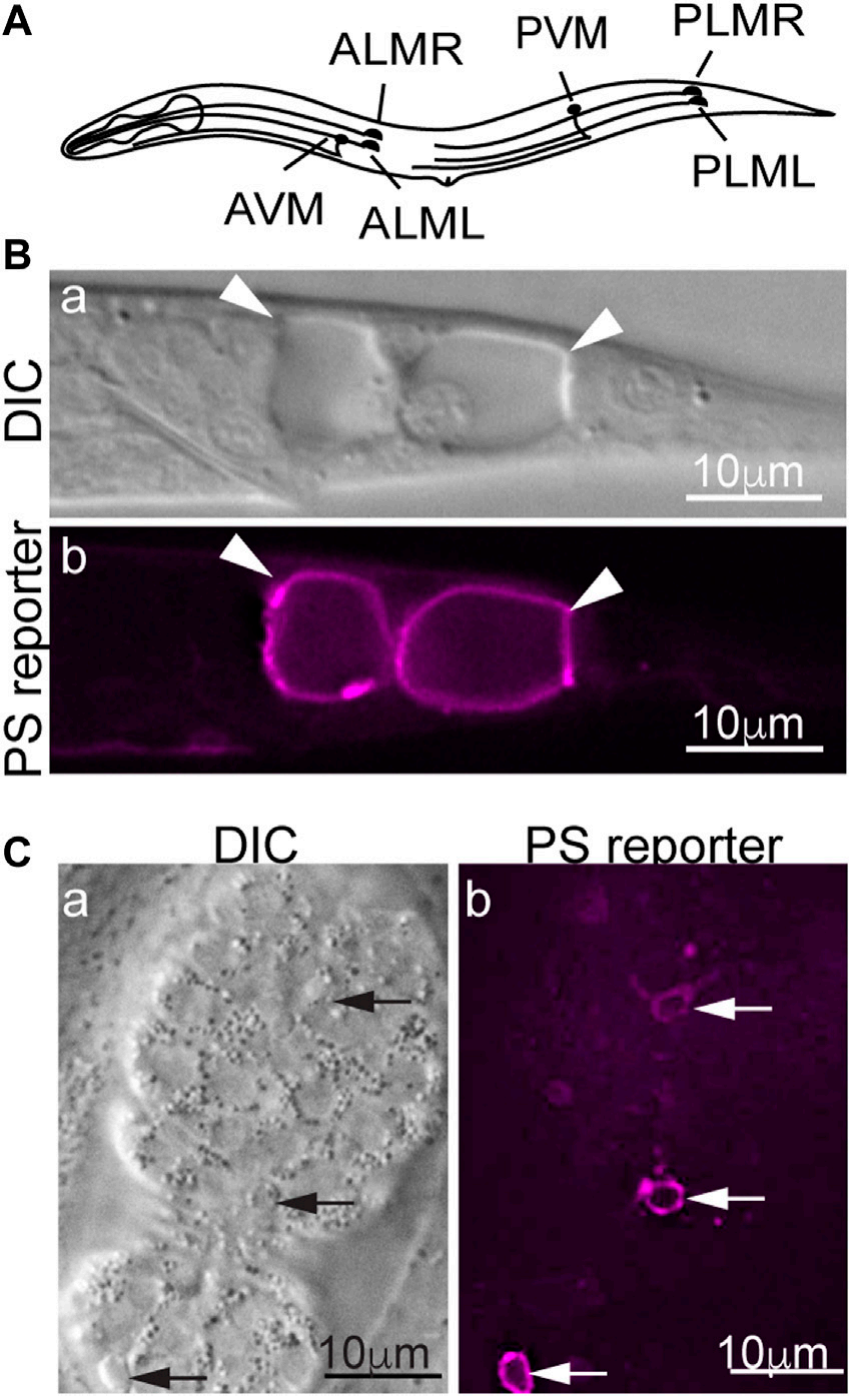
PS is detected on the surfaces of necrotic and apoptotic cells in *Caenorhabditis elegans*. MFG-E8 is a high-affinity PS-binding protein. Secreted MFG-E8mKate2 (the PS reporter) interacts explicitly with PS exposed on cell surfaces. **(A)** A diagram showing the positions and names of the six *Caenorhabditis elegans* touch neurons. **(B)** Differential Interference Contrast (DIC) and epifluorescence images of the tail of a *mec-4(d)* L1-stage larva carrying the MFG-E8::mKate2 reporter. Arrowheads mark the necrotic PLML and PLMR neurons. **(C)** DIC and epifluorescence images of a mid-stage embryo expressing MFG-E8::mKate2. Arrows indicate three apoptotic cells that expose PS.

In the nematode *C. elegans*, dominant (*d*) mutations in specific subunits of the DEG/ENaC super-family of sodium channels, Ca^2+^ channels, and trimeric G proteins induce specific neurons to undergo excitotoxic necrosis (Vlachos and Tavernarakis, 2010). In particular, dominant mutations in *mec-4,* which encodes a core subunit of a multimeric DEG/ENaC sodium channel expressed explicitly in the six mechanosensory (touch) neurons (Figure 1A), trigger the necrosis of these neurons during embryogenesis (Chalfie and Sulston, 1981; Driscoll and Chalfie, 1991). In *mec-4(d)* mutants, necrotic neurons swell to many times their original sizes and are easily distinguishable from living or apoptotic cells under Differential Interference Contrast (DIC) microscope (Figures 1B, C) (Chalfie and Sulston, 1981; Hall et al., 1997). Unlike apoptosis, *mec-4(d)*-induced necrosis does not require the CED-3 caspase activity (Ellis and Horvitz, 1986). Instead, the MEC-4(d) mutations render the mechanosensory Na^+^ channel permeable to Ca^2+^ by altering the channel conformation and in this manner, induce touch neurons to undergo excitotoxic necrosis (Driscoll and Chalfie, 1991; Bianchi et al., 2004). PS on the surfaces of necrotic cells can be detected by a secreted, fluorescently-tagged MFG-E8 reporter, a high-affinity and high-specificity PS-binding protein (Figure 1B) (Hanayama et al., 2002; Li et al., 2015). In addition to the touch neurons, other sensory neurons, motor neurons, and interneurons that undergo necrosis also expose PS, indicating that PS exposure is not a cell type-specific phenomenon (Furuta et al., 2021). That PS is detected on the surfaces of both the necrotic and apoptotic cells indicates that PS could serve as a common “eat me” signal for cells that die of different mechanisms to attract engulfing cells. Indeed, in *C. elegans*, the PS molecules on the necrotic and apoptotic cells' surfaces are recognized by the phagocytic receptor CED-1 localized on the plasma membrane of neighboring hypodermal cells, which subsequently engulf these cells (Li et al., 2015).

Similarly, in mammals, PS has been reported to serve as an “eat me” signal for necrotic cells, although other “eat me” signals that attract phagocytes to necrotic cells, including the complement factors and histidine-rich glycoproteins, were also reported (Gaipl et al., 2001; Böttcher et al., 2006; Poon et al., 2010a). Please see (Krysko et al., 2006b; Westman et al., 2019), two excellent reviews for detailed descriptions of these other “eat me” signal molecules for necrotic cells. Reports have demonstrated that PS is externalized on the surfaces of necrotic mouse hybridoma cells induced by heat (Cocco and Ucker, 2001), necrotic human peripheral blood lymphocytes induced by heat (Böttcher et al., 2006), and necrotic mouse thymocytes induced by heat or H_2_O_2_ (Budai et al., 2019), all of which eventually attract macrophages. Various cells undergoing necroptosis, a death receptor-mediated programmed necrosis, including mouse fibrosarcoma cells, mouse embryonic fibroblast, human myelomonocyte, human keratinocyte, and mouse bone marrow-derived macrophage also externalize PS (Brouckaert et al., 2004; Gong et al., 2017; Zargarian et al., 2017). In addition, cells undergoing other types of regulated necrosis, such as pyroptosis and ferroptosis, also expose PS on their surfaces (de Vasconcelos et al., 2019; Klöditz and Fadeel, 2019). PS exposure promotes the engulfment of these cells (Brouckaert et al., 2004; Zargarian et al., 2017). Like in apoptotic cells, the PS receptors Tim-4, α_V_β_3_ integrin, and the TAM receptors were reported to mediate the PS-dependent phagocytosis of necrotic mouse thymocytes (Budai et al., 2019).

## The mechanisms of PS exposure on the surfaces of apoptotic cells

Molecular mechanisms of PS exposure of apoptotic cells have been extensively reviewed previously (Segawa and Nagata, 2015; Nagata et al., 2020). This article will briefly describe the findings reported in the literature. In mammalian living cells, PS is maintained almost exclusively in the inner leaflet of the plasma membrane, at least partly by the flippases (also called aminophospholipid translocases), the P4-type ATPases ATP11A and ATP11C, which flip PS from the outer to the inner leaflet in an ATP-dependent manner (Segawa et al., 2014; Segawa et al., 2016). In addition, CDC50A, a chaperon protein, plays an essential role in ensuring the plasma membrane localization and activities of ATP11A and ATP11C (Segawa et al., 2014; Segawa et al., 2016). CDC50A also acts as a chaperon for nine additional P4-type ATPases (Shin and Takatsu, 2020). ATP11C facilitates phospholipids translocation in all leukocytes (Yabas et al., 2016). ATP11C knockout mice display severe B-cell deficiency due to the remaining of PS on the surfaces of B cell precursors, which stimulates the engulfment of pre-B cells by phagocytes (Yabas et al., 2011; Segawa et al., 2018). This defect demonstrates that the asymmetrical enrichment of PS in the inner leaflet of the plasma membrane is essential for protecting cells from phagocytes.

**FIGURE 2:**
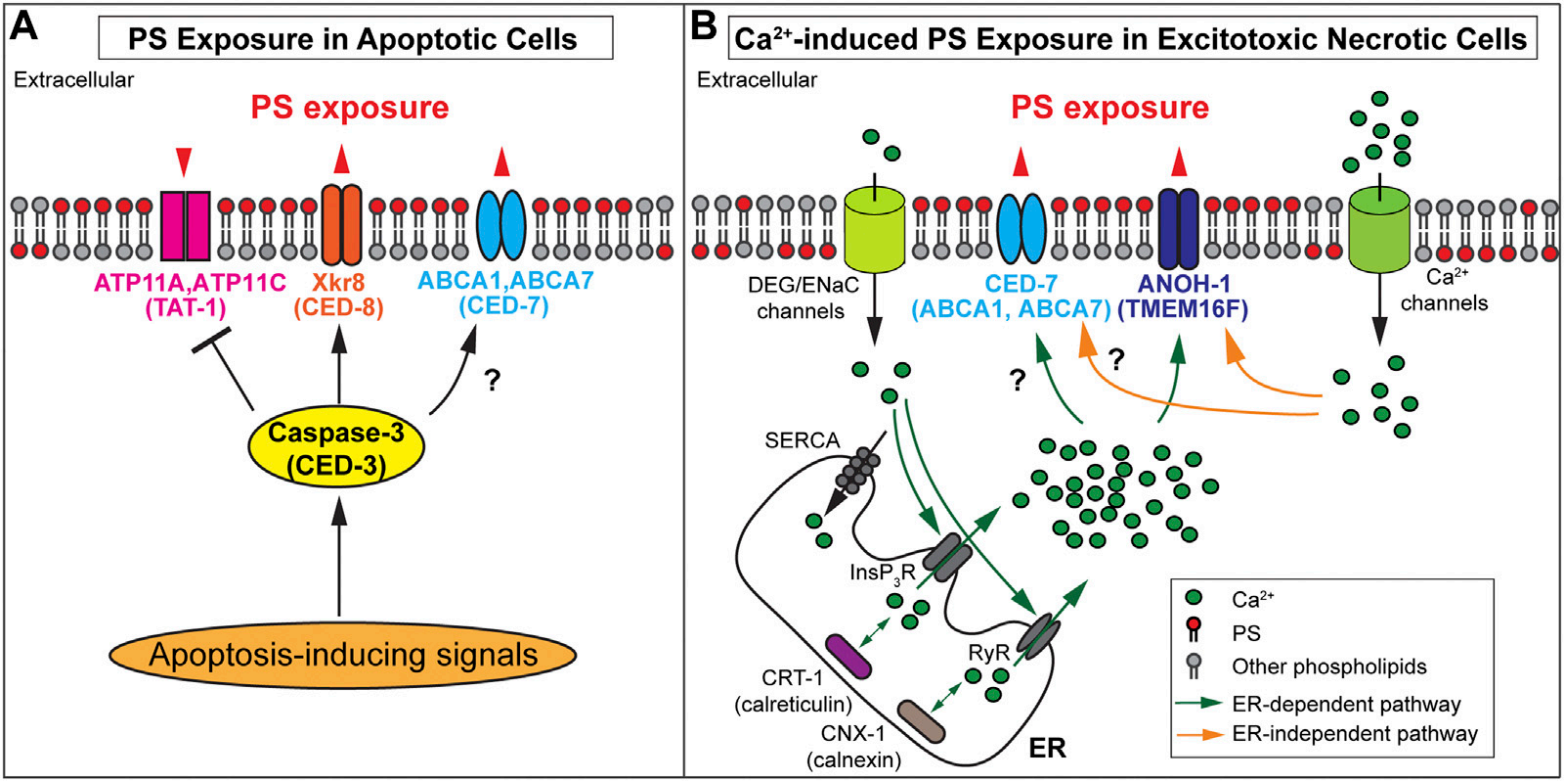
Mechanisms of PS exposure on apoptotic cells and necrotic cells **(A)** Protein names in paratheses are the *Caenorhabditis elegans* homologs of the mammalian proteins. In apoptotic cells, activated caspase-3 cleaves PS scramblases Xkr8 or CED-8 to trigger PS scrambling activity and cleaves the flippases ATP11A and ATP11C to inactivate the PS flipping activity. It is unclear whether the *Caenorhabditis elegans* homolog TAT-1 is inactivated by CED-3. Furthermore, ABCA1 and CED-7 are necessary for PS exposure on apoptotic cells. The black question mark indicates whether the caspase regulates these transporters remains elusive. **(B)** Protein names in paratheses are the mammalian homologs of the *Caenorhabditis elegans* proteins. In excitotoxic necrotic cells, Ca^2+^ influx through various ion channels elevates cytoplasmic Ca^2+^ levels in ER-dependent or ER-independent mechanisms. InsP_3_R (InsP_3_ receptor) and RyR (Ryanodine receptor) are ER Ca^2+^- release channels. SERCA (Sarco-Endoplasmic Reticulum Ca^2+^ ATPase) is a Ca^2+^ reuptake pump. Two ER chaperons, calreticulin, and calnexin are essential in establishing the Ca ^2+^ pool in the ER. The increased cytoplasmic Ca^2+^ is proposed to interact with ANOH-1 or TMEM16F to activate their PS scramblase activities. In addition, CED-7 is necessary to induce PS exposure in excitotoxic necrotic cells. However, it remains unknown whether its mammalian homolog(s) plays the same role and whether these transporters are regulated by cytoplasmic Ca^2+^. **(A-B)** Note that the biochemical activities of C. elegans TAT-1, CED-1, and ANOH-1 have not been examined.

In mouse W3 cells and human Jurkat cells induced to undergo apoptosis, activated caspase 3 has been reported to cleave and inactivate ATP11A and ATP11C (Segawa et al., 2014) (Figure 2A). However, mere inactivation of these flippases is not sufficient for maintaining a substantial level of PS exposure on the surfaces of apoptotic cells. Phospholipid scramblases, which bidirectionally translocate phospholipids between the two membrane leaflets (Bevers and Williamson, 2016), play active roles in exposing PS to the outer surfaces of apoptotic cells in both mammals and *C. elegans*. Suzuki et al. (2013) have identified the essential function of XK-related protein 8 (Xkr8), a phospholipid scramblase, in the exposure of PS on the surfaces of apoptotic cells from multiple human and mouse cell lines. Xkr8 has a caspase-recognition site that is cleaved by the activated mammalian caspase 3 in the cells undergoing apoptosis; this cleavage event activates the scramblase activity of Xkr8 (Suzuki et al., 2013) (Figure 2A). A recent study indicates that in addition to caspase cleavage, phosphorylation of XKr8 also promotes PS exposure (Sakuragi et al., 2019). Studies of Xkr8 knockout mice found that Xkr8 mediates the exposure of PS on the surfaces of apoptotic lymphocytes and aged neutrophils; furthermore, the lack of Xkr8 results in lupus-like autoimmune disease due to the defect of the clearance of apoptotic lymphocytes and aged neutrophils, indicating that the lack of PS exposure on apoptotic cells perturbed their removal (Kawano and Nagata, 2018). In mammalian apoptotic cells, in addition to Xkr8, the mammalian ATP Binding Cassette Subfamily A Members 1 and 7 (ABCA1 and ABCA7) transporters contribute another PS externalization activity (Alder-Baerens et al., 2005; Quazi and Molday, 2013). Both ABCA1 and ABCA7 facilitate the clearance of apoptotic cells (Hamon et al., 2000; Jehle et al., 2006), although controversial results have also been reported (Williamson et al., 2007). So far, whether ABCA1 and ABCA7 transporters are regulated by caspase activity remains elusive.

PS exposure of apoptotic cells was also extensively studied in *C. elegans*, and most key molecular mechanisms are evolutionarily conserved. *Caenorhabditis elegans* have a close homolog for each of the mammalian ATP11A (TAT-1) (Darland-Ransom et al., 2008), Xkr8 (CED-8) (Stanfield and Horvitz, 2000), and ABCA1 (CED-7) (Wu and Horvitz, 1998a). These proteins were reported to function similarly to their mammalian homologs in regulating the exposure of PS on cell surfaces, that is, CED-8 and CED-7 promote PS exposure (Venegas and Zhou, 2007; Chen et al., 2013; Suzuki et al., 2013), whereas TAT-1 is proposed to maintain the asymmetric distribution of PS in the plasma membrane of living cells (Darland-Ransom et al., 2008). Both TAT-1 and CHAT-1, the *C. elegans* homolog of CDC50A, are required for the PS distribution to the cytoplasmic side of intracellular vesicles in the endocytosis pathway, yet the role of CHAT-1 in the PS exposure on plasma membrane has not been tested (Chen et al., 2010). The cleavage of CED-8 by CED-3, the *C. elegans* ortholog of caspase 3, is reported to activate the PS-exposure activity of CED-8 (Chen et al., 2013; Suzuki et al., 2013). Whether CED-3 activates CED-7 and inactivates TAT-1 through protease cleavage remains to be examined. In addition, an independent report indicates that knocking down *tat-1 via* RNA interference abrogates PS exposure instead of facilitating it (Zullig et al., 2007), casting some doubt on the proposed role of TAT-1 in suppressing PS exposure on living cells.

## Cytoplasmic Ca^2+^ induces PS exposure on excitotoxic necrotic cells

### PS is exposed on the surfaces of necrotic cells before cell rupture

Despite the common belief that PS is detected on the surfaces of necrotic cells due to the rupture of necrotic cell membranes (Atkin-Smith, 2021), the *C. elegans* necrotic neurons expose PS while maintaining the integrity of the plasma membrane (Li et al., 2015; Furuta et al., 2021). They are subsequently engulfed as intact cells (Hall et al., 1997; Li et al., 2015). These facts suggest that necrotic cells are able to expose PS actively. The PS exposure on necroptotic mammalian fibroblast cell lines (NIH 3T3 and L929) occurs preceding the loss of the plasma membrane integrity, again indicating that PS is actively exposed on cell surfaces before membrane disruption (Gong et al., 2017; Zargarian et al., 2017).

### A mechanism that is dependent on the release of Ca^2+^ from the endoplasmic reticulum

Caspase activity is not necessary for inducing excitotoxic necrosis in *C. elegans* (Ellis and Horvitz, 1986; Chalfie and Wolinsky, 1990; Nagarajan et al., 2014); In excitotoxic necrosis, studies indicate that the elevation of intracellular Ca^2+^ levels plays a vital role in the induction of necrosis (Xu et al., 2001; Berliocchi et al., 2005; Celsi et al., 2009; Bano and Ankarcrona, 2018). Electrophysiological studies revealed that the MEC-4(d) mutations in *C. elegans* alter the conformation of the mechanosensory Na^+^ channel (of which MEC-4 is a subunit), allowing Ca^2+^ to enter the cytoplasm through this channel (Bianchi et al., 2004). By monitoring the signal intensity of an *in vivo* Ca^2+^ reporter specifically expressed in touch neurons in the developing *mec-4(d)* mutant embryos, it was observed that the cytoplasmic Ca^2+^ level increases in the touch neurons before cell swelling, a feature of necrosis, prior to PS exposure on necrotic cell surfaces Furuta et al., (2021); Furuta and Zhou (2021). Quantitative analysis found a strong correlation between the levels of cytoplasmic Ca^2+^ and the levels of PS exposure. The endoplasmic reticulum (ER) is a major intracellular Ca^2+^ storage pool (Sammels et al., 2010). The release of Ca^2+^ from the ER to the cytoplasm is essential for the induction of necrosis in *mec-4(d)* mutants (Xu et al., 2001). Inactivation of a major ER Ca^2+^-release channel, or an ER-located Ca^2+^-chaperon essential for the establishment of the Ca^2+^ pool inside the ER lumen, greatly reduces the level of PS exposed on the surfaces of necrotic cells in dominant mutant strains of *mec-4*, *unc-8*, and *deg-1*, each of which encodes a different subunit of the DEG/ENaC channel (Furuta et al., 2021). Furthermore, artificially increasing the cytoplasmic Ca^2+^ level through inactivating Sarco-Endoplasmic Reticulum Ca^2+^ ATPase (SERCA), the Ca^2+^ reuptake pump on the ER membrane (Treiman et al., 1998; Doan et al., 2015), induces both necrosis and PS exposure on necrotic cells (Furuta et al., 2021). In addition, inhibition of downstream necrotic events blocks the morphological changes of touch neurons but not the PS exposure (Furuta et al., 2021). Together, the above results indicate that 1) the increase of cytoplasmic Ca^2+^ induces both necrosis and PS exposure, 2) The Ca^2+^ leaked into touch neurons through the mutant DEG/ENaC channel in the *mec-4(d)*, *unc-8(d)*, or *deg-1(d)* mutant strains is necessary but not sufficient to induce PS exposure or necrosis; instead, the Ca^2+^-induced Ca^2+^ release from the ER, which further elevates the cytoplasmic Ca^2+^ level, is necessary for the induction of PS exposure and necrosis, and 3) the induction of PS exposure is independent of the induction of necrosis (Figure 2B). These results further implicate the existence of different proteins that act to induce various cellular events, such as PS exposure and cell swelling, in response to the elevated level of cytoplasmic Ca^2+^.

### An ER-independent and Ca^2+^-dependent PS exposure mechanism

A dominant, constitutively active mutation of *deg-3,* which encodes a ligand-gated calcium channel belonging to the nicotinic acetylcholine receptor family (Treinin and Chalfie, 1995), like *mec-4(d)* mutations, results in the excitotoxic necrosis of neurons (Chalfie and Wolinsky, 1990). However, unlike *mec-4(d)* mutations, this mutation induces necrosis and PS exposure in a manner independent of the contribution of the ER Ca^2+^ pool (Xu et al., 2001; Furuta et al., 2021). The DEG-3 Ca^2+^ channel has high Ca^2+^ permeability, and the *deg-3(u662)* dominant mutation causes this channel to remain open constitutively (Treinin et al., 1998). In addition to *deg-3(d)*, a dominant mutation in *trp-4,* which encodes a transient receptor potential (TRP) channel, another Ca^2+^ channel (Li et al., 2006), also induces the necrosis of neurons and PS exposure (Nagarajan et al., 2014). Interestingly, although *trp-4(d)*-induced necrosis is dependent on the ER Ca^2+^ release (Nagarajan et al., 2014), *trp-4(d)*-induced PS exposure is not (Furuta et al., 2021). TRP channels are highly permeable to Ca^2+^ (Gees et al., 2010). The TRP-4(d) mutation is likely to keep the Ca^2+^ channel in a constitutively open state. Together, the differential requirements for the ER contribution to PS exposure observed from the constitutively open DEG/ENaC channel and Ca^2+^ channels suggest that the critical factor that triggers PS exposure is the cytoplasmic Ca^2+^ level. When a mutant Ca^2+^ channel allows constitutive and high permeability influx of Ca^2+^ into neurons, the contribution of the Ca^2+^ from the ER pool is not necessary; on the other hand, when a mutant ion channel only allows a trickling amount of Ca^2+^ to enter a neuron, the “Ca^2+^-induced Ca^2+^ release” from the ER becomes essential for inducing PS exposure.

It is worth noting that a few previous studies indicated the role of Ca^2+^ in inducing PS exposure on apoptotic cells (Hampton et al., 1996; Verhoven et al., 1999; Zhivotovsky and Orrenius, 2011). On the other hand, lines of evidence demonstrate against the involvement of Ca^2+^ in PS exposure on the surfaces of apoptotic cells (Hampton et al., 1996; Schoenwaelder et al., 2009). In *C. elegans*, Furuta et al. (2021) demonstrated that impairing the release of ER Ca^2+^ into the cytoplasm did not affect the level of the PS on the surfaces of apoptotic cells. In conclusion, two different kinds of mechanisms regulate PS exposure: apoptotic cells utilize Ca^2+^-independent PS exposure driven by caspase activity, while necrotic cells utilize Ca^2+^-dependent mechanisms (Figure 2).

## Scramblase and flippasses that play roles in Ca^2+^-dependent PS exposure on excitotoxic necrotic cells

Mammalian platelets activated during blood coagulation expose PS in response to Ca^2+^ influx but do not undergo cell death (Bevers et al., 1983; Dachary-Prigent et al., 1995). On the other hand, when platelets are induced to undergo apoptosis, they expose PS in a caspase-dependent yet Ca^2+^-independent manner (Schoenwaelder et al., 2009). These results again demonstrate the existence of both the Ca^2+^-dependent and -independent PS exposure mechanisms in platelets. Mammalian TMEM16F is a high-affinity Ca^2+^-binding protein and a Ca^2+^-dependent scramblase that is responsible for exposing PS when platelets are activated (Suzuki et al., 2010; Fujii et al., 2015). A mutation in TMEM16F was reported to cause Scott syndrome, a human bleeding disorder (Suzuki et al., 2010), demonstrating the essential role of human TMEM16F in blood clotting.

ANOH-1 is the *C. elegans* homolog of mammalian TMEM16F (Wang et al., 2013; Li et al., 2015). It is specifically expressed in neurons and presented on the plasma membrane of the excitotoxic necrotic touch neuron (Li et al., 2015). The amino acid sequences essential for the Ca^2+^-dependent PS scrambling activity identified in mammalian and the fungus *Nectria haematococca* TMEM16F (Suzuki et al., 2010; Brunner et al., 2014) are conserved in *C. elegans* ANOH-1 (Li et al., 2015). Deletion in the *anoh-1* gene causes the reduction of PS exposure on neurons undergoing necrosis induced by the *mec-4(d)* and *trp-4(d)*, suggesting that the Ca^2+^-induced PS exposure is dependent on ANOH-1 (Li et al., 2015; Furuta et al., 2021) (Figure 2B). In *anoh-1(−)*; *mec-4(d)* double mutant larvae, necrotic touch neurons linger much longer before being engulfed, presumably as a result of the reduction of PS exposure activity (Li et al., 2015). On the other hand, *anoh-1* deletion does not affect the exposure of PS on apoptotic cells (Li et al., 2015). Although its biochemical activity has not been determined, *C. elegans* ANOH-1 is proposed to act as a Ca^2+^-dependent phospholipid scramblase specific for necrotic cells (Li et al., 2015; Furuta et al., 2021).

Li et al. (2015) identified not only *C. elegans* ANOH-1 but also another *C. elegans* protein that promotes PS exposure on necrotic neurons. This protein is CED-7, the *C. elegans* homolog of ABCA1 transporter (Li et al., 2015). In *ced-7* loss-of-function mutants, PS exposure on both apoptotic and necrotic cells are greatly reduced in *C. elegans* (Venegas and Zhou, 2007; Li et al., 2015). In *ced-7; anoh-1* double mutants, the PS exposure defect is more severe than in each single mutant, and the necrotic touch neurons persist for much longer before being engulfed than in each of the *anoh-1* or *ced-7* single mutants, indicating that ANOH-1 and CED-7 act in parallel to promote PS exposure and the engulfment of necrotic cells (Li et al., 2015). *ced-7* mutant phenotypes indicate that a common molecular mechanism that involved CED-7 is needed in both apoptotic and necrotic cells for efficient PS exposure. It is unclear whether *C. elegans* CED-7 is regulated by Ca^2+^ in necrotic cells (Figure 2B). Whether mammalian ABCA1 and ABCA7 play any role in the PS exposure on necrotic cells also awaits to be tested.

In addition to the scramblase activity, Ca^2+^ might also inactivate flippase(s) to “maintain” the exposed PS on the surface of necrotic cells. Segawa et al. (2018) reported that the PS exposed on the surfaces of lymphoma cells induced by Ca^2+^-ionophore is quickly internalized when the ionophore is removed. Yet, in ATP11A^−/−^ATP11C^−/-^ double deletion cells, PS remains on the outer leaflet persistently. These observations indicate that cytoplasmic Ca^2+^ regulates flippase activities. Together, all the above information indicates that in necrotic cells, the increase of the cytoplasmic Ca^2+^ level might activate scramblase(s) and inactivate flippase(s) simultaneously to achieve continuous PS exposure. The mechanisms of how cytoplasmic Ca^2+^ induces PS exposure on necrotic cells need much further investigation.

## Ca^2+^-independent PS-exposure on the surfaces of necrotic cells

Interestingly, a recent study conducted in TNF-induced necroptotic mouse embryonic fibroblast cells has found that although both Ca^2+^ influx and PS exposure occur when cells undergo necroptosis, PS exposure does not require either Ca^2+^ influx or caspase activity (Gong et al., 2017). This study further implicates that mixed lineage kinase-like (MLKL), which mediates membrane disruption in necroptosis, directly causes the exposure of PS. Another study showed that activated MLKL promotes PS exposure on interferon (IFN)-ɣ induced necroptotic mouse embryonic fibroblast cells and human colorectal adenocarcinoma cells (Chen et al., 2019), supporting the PS exposure-promoting role of MLKL in necroptotic cells.

In another example, a high dose of extracellular ATP induces PS exposure on mammalian T cells and other cell types and the eventual necrosis (Taylor et al., 2008; Ryoden et al., 2022). ATP causes these effects through binding to P2X7, an ATP-gated non-selective cation channel (Ryoden and Nagata, 2022). Activation of P2X7 was reported to cause Ca^2+^ influx (North, 2002; Ousingsawat et al., 2015). Furthermore, the Ca^2+^-dependent scramblase TMEM16F was reported to promote PS exposure in response to ATP in the HEK293 cells (Ousingsawat et al., 2015). However, a recent study reported that TMEM16F is dispensable for the P2X7-mediated PS exposure in a WR19L mouse lymphoblast-derived cell line; instead, Xk, a paralogue of the Xkr8 scramblase, and VPS13A, a cytoplasmic lipid transporter, are necessary for this event (Ryoden et al., 2022). Therefore, whether Ca^2+^ is required for this type of PS exposure is a matter of debate that needs further investigation. These studies nevertheless suggest that the requirement of Ca^2+^ for PS exposure might depend on how the necrosis is induced and the types of cells that undergo necrosis.

## Summary

Here we summarize a few features behind the molecular mechanisms that regulate PS exposure on cell surfaces. First, cells die of apoptosis and necrosis, two different death mechanisms, expose the same “eat me” signal–PS and likely attract the same phagocytic receptor(s). For example, in *C. elegans*, the phagocytic receptor CED-1 recognizes both apoptotic and necrotic cells and initiates the engulfment of both kinds of dying cells. However, the upstream mechanisms that promote PS exposure are strikingly different between apoptotic and necrotic cells. Whereas apoptotic cells rely on caspase-mediated PS-exposure mechanisms, excitotoxic necrosis depends on a high level of cytoplasmic Ca^2+^ to induce PS exposure. The activation of different scramblases during apoptosis and necrosis discovered in *C. elegans* demonstrated the differential molecular mechanisms of PS exposure. On the other hand, *C. elegans* CED-7 facilitates PS exposure on both apoptotic and necrotic cells, suggesting that either CED-7 provides a basal-level, constitutive PS-exposure activity that is counteracted by the PS flippase(s) in living cells, or that CED-7 can be activated by multiple upstream signals. Nevertheless, both common and differential mechanisms are utilized to regulate PS exposure on dying cell surfaces. Interestingly, necroptotic cells and certain types of cells induced to undergo necrosis by ATP seem to apply specific Ca^2+^-independent mechanisms for PS exposure. Necrosis can be induced by various kinds of stimuli. The detailed mechanisms of PS exposure, which might be stimuli-specific and cell type-specific, await further investigation. Further dissecting the PS exposure mechanisms would help us understand the cell clearance mechanisms, especially in the disease context, and may serve as a critical step for future therapeutic intervention.
